# Dual-task costs while walking increase in old age for some, but not for other tasks: an experimental study of healthy young and elderly persons

**DOI:** 10.1186/1743-0003-5-27

**Published:** 2008-11-13

**Authors:** Otmar Bock

**Affiliations:** 1 Institute of Physiology and Anatomy, German Sport University, Köln, Germany

## Abstract

**Background:**

It has been suggested in the past that the ability to walk while concurrently engaging in a second task deteriorates in old age, and that this deficit is related to the high incidence of falls in the elderly. However, previous studies provided inconsistent findings about the existence of such an age-related dual-task deficit (ARD). In an effort to explain this inconsistency, we explored whether ARD while walking emerges for some, but not for other types of task.

**Methods:**

Healthy young and elderly subjects were tested under five different combinations of a walking and a non-walking task. The results were analysed jointly with those of a previous study from our lab, such that a total of 13 task combinations were evaluated. For each task combination and subject, we calculated the mean dual-task costs across both constituent tasks, and quantified ARD as the difference between those costs in elderly and in young subjects.

**Results:**

An analysis of covariance yielded no significant effects of obstacle presence and overall task difficulty on ARD, but a highly significant effect of visual demand: non-walking tasks which required ongoing visual observation led to ARD of more than 8%, while those without such requirements led to near-zero ARD. We therefore concluded that the visual demand of the non-walking task is critical for the emergence of ARD while walking.

**Conclusion:**

Combinations of walking and concurrent visual observation, which are common in everyday life, may contribute towards disturbed gait and falls during daily activities in old age. Prevention and rehabilitation programs for seniors should therefore include training of such combinations.

## Introduction

Human gait deteriorates in old age. Walking speed and the stability of the walking pattern decrease [1-3], and the incidence of falls increases dramatically: about 25% of the 70 year olds, 35% of the 75 year olds, and 50% of the over 80 year olds fall at least once per year [4-6]. Many of these falls don't result in physical injury, but they often have negative psychosocial consequences such as fear of falling, self-imposed inactivity, dependence on others [7], and ultimately, admittance into nursing homes [8]. To counteract this downward spiral, it is important to understand the reasons why locomotion is degraded in the elderly and, based on this understanding, to develop efficient prevention and rehabilitation programs.

Previous studies proposed various explanations for gait impairments in old age, such as reduced sensory functions, muscle weakness, and slowdown of psychomotor processing [reviews in [7,9,10]], as well as a reduced ability to perform two tasks concurrently [11,12]. Our present work focuses on the latter explanation. According to this view, elderly persons are at a particular risk of falling when they move through their home while talking to a friend on the phone, walk down a street while mentally rehearsing the shopping list, cross a roadway while watching for traffic, etc. Indeed, a number of studies provided experimental evidence that seniors have more problems than younger persons to perform two tasks concurrently [13-16]. This age-related dual-task deficit (ARD) has been attributed to the shrinkage of prefrontal brain areas in old age [17-19], since those areas are strongly related to executive functions – such as the management of multiple-tasks [17,20].

Most previous studies documented ARD using tasks which required manual and/or verbal responses; their findings are therefore not necessarily generalizable to locomotion. Other authors included a task which required a *postural *response, such as maintenance of steady stance [21-23], or recovery of stance after a perturbation [24,25]; those authors observed ARD as well. Yet other work included *walking *as a task, but unfortunately, the resultant data are inconclusive. Some of the latter studies compared single- and dual-task performance on only one of the two concurrent tasks, and thus confounded ARD with task priority: a larger dual-task decrement of seniors on the registered task may not reflect ARD, but rather seniors' higher priority for the non-registered task [26]. Other authors avoided this design flaw, but yielded discrepant results: some observed *no *ARD while walking [27,28], while others reported *substantial *ARD while walking [29,30]. This discrepancy is probably not explainable by between-study differences of task difficulty, since ARD is *un*related to the difficulty of walking and non-walking tasks [13,29,30]. The emergence of ARD while walking therefore seems to depend on some *specific *task characteristics, present only in a part of the above studies.

In search for those characteristics, our group has recently compared eight different combinations of a walking and a non-walking task [31], and found ARD for only one of them. This combination differed from the other ones in three respects: subjects had to walk on a treadmill rather than on solid ground, they had to avoid obstacles while walking, and had to engage in ongoing visual observation of the non-walking task. It remained open in the above study which of these differences was responsible for the emergence of ARD, and the present work was therefore designed to find out.

## Methods

Eighteen younger (24.3 ± 3.5 years of age, 9 female and 9 male) and fifteen older subjects (67.2 ± 3.6 years of age, 7 female and 8 male) participated in Exp. A. Sixteen younger (22,4 ± 1.6 years of age, 6 female and 10 male) and sixteen older subjects (66.1 ± 3.7 years of age, 6 female and 10 male) participated in Exp. B. All elderly subjects lived independently in the community, and exhibited no signs of cognitive or sensorimotor deficits except corrected vision and hearing. No subject had been involved in sensorimotor research before. All subjects signed an informed consent statement before participating in this study, which was pre-approved by the author's Ethics committee.

Experiment A was designed to find out whether the use of a treadmill was essential for the emergence of ARD in our previous study. Furthermore, we wanted to find out whether ongoing visual observation but not visual memory was crucial. Subjects therefore walked on solid ground while avoiding obstacles, engaged in a visual checking task, and/or kept a visual scene in memory. The walking and each non-walking task were administered separately as well as concurrently.

For task *walk*_*o*_, an obstacle parcours was laid out in a 2.2 m wide hallway. Paper sheets of 60 cm width and 21 cm length were distributed along the floor at center-to-center distances of 1.8*λ, 3.5* λ, 5.5* λ, 3.5* λ, 1.5* λ, 5.5* λ, and 1.5* λ, where λ denotes the mean step length of a given subject, as determined prior to the experiment. We found in preliminary tests that this obstacle layout is complex enough to disturb the gait rhythm, but simple enough to be negotiated by elderly persons without help. Subjects started to walk two steps in front of the first obstacle, and finished one step behind the last. They walked at their preferred speed, and all succeeded in not touching the obstacles. We quantified their performance as mean walking speed from the last footfall before the second obstacle until the first footfall after the last obstacle.

In task *check*_*gw*_, subjects held a clipboard in their left, and a pen in their right hand. A paper sheet on the clipboard displayed pairs of boxes, arranged in three columns of 25 rows. One box of each pair was grey and the other white, and their order (grey-white versus white-grey) varied randomly between pairs. A new paper sheet with a different order of pairs was used for each task repetition. Subjects were instructed to scan the paper from top to bottom, column by column, and to check off the grey box of the first pair, the while box of the second, the grey box of the third, the white box on the fourth, etc. We quantified their performance as the number of boxes checked correctly within 20 s of quiet stance (single-task condition), or during negotiation of the obstacle parcours (dual-task condition).

In task *memo*, subjects inspected for 20 s a drawing which showed a familiar scene, such as children at play. Afterwards, they stood still for 20 s (single-task condition) or negotiated the obstacle parcours (dual-task condition), and were then asked ten questions about the drawing such as "how many toy trucks did you see?". Their performance was scored as number of correct responses. A new drawing was used for each task repetition.

Each subject participated in the single-task conditions *walk*_*o*_, *check*_*gw*_, and *memo*, and in the dual-task conditions *walk*_*o*_+*check*_*gw*_, and *walk*_*o*_+*memo*. Each condition was repeated three times, and the average score across repetitions was used for further analyses. The order of conditions varied randomly between subjects. The experiment took about 30 minutes, including instructions and other preliminary activities.

Experiment B was designed to find out whether the emergence of ARD depended on the use of obstacles in *walk*_*o*_, and/or on rule switching in *check*_*gw*_. We therefore administered the additional tasks *walk*, where subjects walked down an obstacle-free hallway at preferred speed for the same distance as in Exp. A, and *check*_*g*_, where subjects checked off just the grey boxes in all grey-and-white pairs. Performance was quantified as the mean walking speed from the second to the second-to-last step, and as the number of boxes checked correctly within 20 s. Each subject participated in two repetitions of *walk, check*_*g*_, *walk*_*o*_, *check*_*gw*_, *walk+check*_*g*_, *walk+check*_*gw*_, *walk*_*o*_+*check*_*g*_, *and walk*_*o*_+*check*_*gw*_, with the order of conditions varying randomly between subjects. The experiment took about 45 minutes, including instructions and other preliminary activities.

## Results

**Figure 1:**
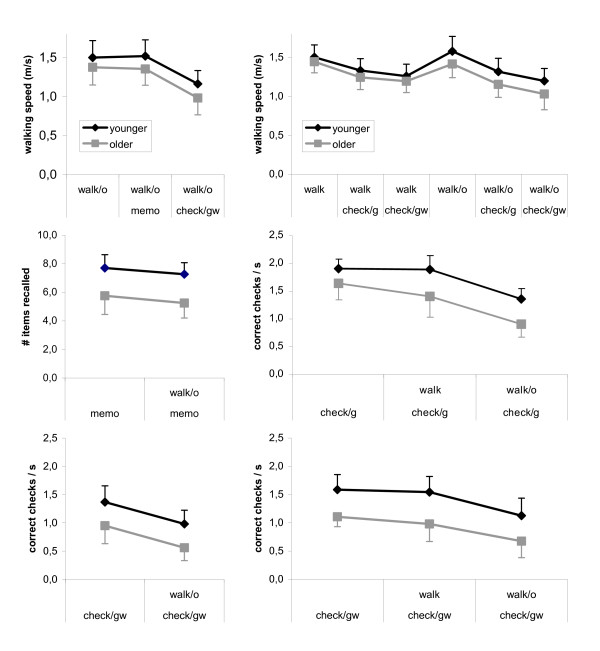
**Subjects' performance on all constituent tasks of Exp. A (left) and B (right)**. Each symbol represents the average score of younger (black) or older (grey) subjects, and each error indicator the corresponding standard deviation.

The left part of Fig. 1 illustrates the outcome of Exp. A. Older subjects performed generally less well than younger ones, in all single- and dual-task conditions. In both age groups, walking speed (top plot) was not affected by task *memo*, but was substantially reduced by task *check*_*gw*_. Memory recall (middle plot) decreased slightly, and checking performance (bottom plot) decreased distinctly when the walking task was added. In accordance with these observations, two-way analyses of variance (ANOVAs) yielded significant effects of the between-factor Age on the dependent variables walking speed (F(1,31) = 9.36; p < 0.01), memory recall (F(1,31) = 40.18; p < 0.001), and checking performance (F(1,31) = 23.88; p < 0.001), as well as significant effects of the within-factor Condition on walking speed (F(2,62) = 147.38; p < 0.001), memory recall (F(1,31) = 7.10; p < 0.05), and checking performance (F(1,31) = 97.26; p < 0.001). The Age*Condition interactions were non-significant for all three dependent variables.

To quantify subjects' ability for executing two tasks concurrently, we calculated for each subject and task the dual-task costs DTC according to the customary formula [32]

(1) DTC [%] = 100 * (single-task score - dual-task score)/single-task score

The outcome is summarized in the top part of Tab. 1. DTC was small for both constituent tasks of *walk*_*o*_+*memo *(i.e., for walking as well as for memorizing), but was large for both constituent tasks of *walk*_*o*_+*check*_*gw*_. Elderly subjects had larger DTC than younger ones, particularly in *walk*_*o*_+*check*_*gw*_, but the difference between age groups failed to reach statistical significance in t-tests (last column of Tab. 1). The latter outcome reflects the lack of a significant Age*Condition interaction in the above ANOVAs.

**Table 1:** Dual-task costs of the constituent tasks in Exp. A and B.

**Exp.**	**task combination**	**task**	**young Ss.**	**elderly Ss**.	**t**
A	walk_o _+ memo	walk_o_	-1.21 ± 5.71	1.22 ± 6.20	1.17^n.s.^
		
		memo	3.84 ± 12.95	6.78 ± 15.91	0.58^n.s.^
	
	walk_o_+check_gw_	walk_o_	22.46 ± 8.58	28.64 ± 11.11	1.80^n.s.^
		
		check_gw_	28.86 ± 11.67	39.44 ± 20.71	1.84^n.s.^

B	walk + check/g	walk	11.20 ± 6.37	13.84 ± 7.87	1.04^n.s.^
		
		check_g_	0.44 ± 12.00	14.53 ± 17.31	2.67*
	
	walk + check_gw_	walk	16.02 ± 8.11	16.96 ± 10.61	0.28^n.s.^
		
		check_gw_	1.34 ± 17.25	10.61 ± 29.00	1.09^n.s.^
	
	walk_o _+ check/g	walk_o_	16.35 ± 7.19	18.34 ± 9.15	0.68^n.s.^
		
		check_g_	28.07 ± 12.82	44.81 ± 10.68	4.01***
	
	walk_o _+ check_gw_	walk_o_	23.83 ± 8.32	26.85 ± 13.80	0.74^n.s.^
		
		check_gw_	28.77 ± 16.36	39.36 ± 24.11	1.45^n.s.^

Data columns indicate the mean ± standard deviation of DTC in young and elderly subjects, and the t-scores of t-tests, with n.s., *, and *** denoting p > 0.05, p < 0.05, and p < 0.001.

Subjects' performance in Exp. B is illustrated in the right part of Fig. 1. Again, older subjects performed generally less well than younger ones. Walking speed was comparable in *walk *and *walk*_*o*_, and decreased somewhat when a second task was added. Checking performance was better in *check*_*g *_than in *check*_*gw*_, decreased slightly when *walk *was added, and more distinctly when *walk*_*o *_was added. In accordance with these observations, two-way ANOVAs yielded significant effects of Age on walking speed (F(1,30) = 5.83; p < 0.05), performance in *check*_*g *_(F(1,30) = 25.36; p < 0.001), and in *check*_*gw *_(F(1,30) = 45.22; p < 0.001). We also found significant effects of Condition on walking speed (F(5,150) = 70.93; p < 0.001), performance in *check*_*g *_(F(2,60) = 106,80; p < 0.001), and in *check*_*gw *_(F(2,60) = 37.21; p < 0.001). All Age*Condition interactions were again non-significant. The corresponding DTC scores are summarized in the bottom part of Tab. 1. They are substantial, except when younger subjects performed one of the checking tasks in combination with obstacle-free walking. Again, elderly subjects had larger DTC than younger ones, but unlike in Exp. A, the group difference now became significant for two task combinations.

The present findings can be compared to those from our previous study [31], thus bringing together data from 13 task combinations, collected in 214 elderly and 205 younger subjects. The tasks used in the previous study are briefly described in Tab. 2. To present the outcome of both studies compactly, we calculated for each subject, and each task combination *taskα + taskβ*, the mean dual-task costs as

**Table 2:** Summary of experimental tasks used in our previous study.

**acronym**	**description**	**dependent variable**
walk	walk at preferred speed down a 2.2 m wide hallway, or along a 0.8 m wide circular path	mean speed

walk_n_	walk at preferred speed along a 0.2 m wide circle	mean speed

walk_nf_	walk at maximum speed along a 0.2 m wide circle	mean speed

treadmill_o_	walk on a treadmill (elderly 0.8, younger 1.2 m/s), while obstacles appear at unknown intervals	percent of obstacles negotiated without contact

spell	spell a word of 18–21 letters	number of correctly spelled letters per 20 s

shape	hear names of 10 geometrical shapes while walking, and repeat them afterwards	number of correctly repeated shapes

button	close nine different buttons on a jacket, open them, close them again, etc.	number of completed button actions per 120 s

detect	press knob when a dot appearing in a random-dot pattern forms a square with three pre-existing dots	percent and RT of hits, percent of correct rejections

(2) $$\text{mean DTC}(task\alpha +task\beta )[\%]=\frac{\text{DTC}\left(task\alpha \right)+\text{DTC}\left(task\beta \right)}{\text{2}}.$$

**Figure 2:**
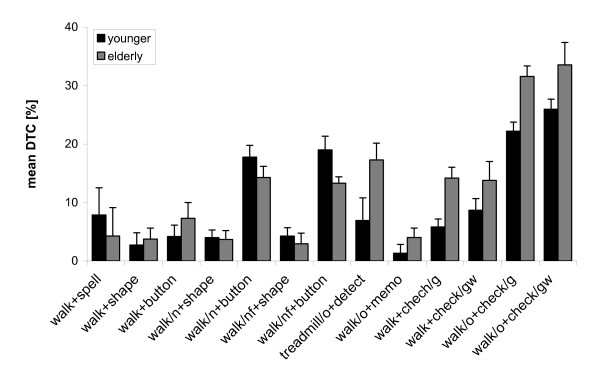
**Mean dual-task costs of all task combinations in our present and previous study**. [31]. Each bar represents the average score of younger (black) or older (grey) subjects, and each pair of bars one task combination. Error indicators are 20% of the corresponding standard deviation. An age-related deficit of dual-task performance exists where grey bars are larger than black bars.

By calculating the costs across both tasks, we can express subjects' dual-task ability irrespective of their individual task priorities [14,33]. The outcome of this calculation is illustrated by the five rightmost pairs of bars in Fig. 2, with each pair representing one combination from Exp. A and B. Since *walk*_*o*_+*check*_*gw *_was administered both in Exp. A and B, the respective data were merged for presentation in Fig. 2 as well as for further analyses.

Mean DTC for the five rightmost task combinations in Fig. 2 were generally higher in elderly than in younger subjects. This age difference was significant in t-tests for *walk + check*_*g *_(t = 2.92, p < 0.01), *walk*_*o*_+*check*_*g *_(t = 3.14, p < 0.01), and *walk*_*o*_+*check*_*gw *_(t = 2.67, p < 0.01), but not *walk*_*o*_+*memo *(t = 0.98, p > 0.05) and *walk+check*_*gw *_(t = 1.07, p > 0.05). Not surprisingly, this pattern of findings on mean DTC is quite comparable to that on task-specific DTC shown in Tab. 2. The only exception is *walk*_*o*_+*check*_*gw*_, where the age effect was significant for mean but not for task-specific DTC; this is so because data from two experiments were merged to calculate mean DTC, which increased the sample size, and thus also increased the power of statistical testing.

The remaining pairs of bars in Fig. 2 illustrate mean DTC for the task combinations in our previous study [31]. Taken together, Fig. 2 shows that mean DTC of both age groups was higher for some task combinations than for others. In particular, mean DTC increased when obstacles were used, and when high precision was required in the non-walking task (*button*). Further from Fig. 2, mean DTC was higher in elderly than in younger subjects for some but not for other task combinations, thus reflecting age-related deficits of dual-task performance (ARD). It was the purpose of the present work to determine whether ARD depends on the presence of obstacles in the walking path and/or on the need for ongoing visual observation in the non-walking task (see Introduction). To find out, we quantified ARD of each elderly subject i and task combination k as

(3) $${\text{ARD}}_{\text{i},\text{k}}[\%]={\text{mean DTC}}_{\text{i},\text{k}}-\bar{{\text{mean DTC}}_{\text{k}}}$$

where $\bar{{\text{mean DTC}}_{\text{k}}}$ is the average across all *younger *subjects in task combination k. The resultant ARD scores were submitted to an analysis of covariance, with the between-factors Obstacles (yes/no) and ongoing Visual Observation (yes/no). The following tasks were deemed to require ongoing visual observation: *detect*, *check*_*g*_, and *check*_*gw*_. To guard against possible effects of overall task difficulty, we included $\bar{{\text{mean DTC}}_{\text{k}}}$ as a covariate. Since the age of elderly subjects differed between task combinations (mean age ranged from 65,0 to 70,7 years), we also included each senior's actual age as a covariate.

The analysis yielded a significant effect only for the factor Visual Observation (F(1,204) = 13.45; p < 0.001), not for Obstacles (F(1,204) = 2.65; p > 0.05), the interaction term (F(1,204) = 0.19; p > 0.05), the covariate Difficulty (F(1,204) = 2.89; p > 0.05), nor the covariate Age (F(1,204) = 0.87; p > 0.05). On the average, task combinations with low visual-observation requirements in the non-walking task had a mean ARD of -0.76%, while those with high visual-observation requirements had a mean ARD of 8.53%.

## Discussion

The purpose of the present study was to compare the dual-tasking ability of young and elderly subjects under different combinations of a walking and a non-walking task, in order to determine which task characteristics favor the emergence of age-related dual-task deficits (ARD). Based on our previous work [31], we postulated that ARD may depend critically on the use of a treadmill for walking, the presence of obstacles in the walking path, and/or the need for ongoing visual observation in the non-walking task (see Introduction).

Our data from Exp. A and B clearly show that a treadmill is not critical, since ARD were significant in three out of five task combinations even though subjects walked on solid ground. The data from both experiments further suggest that the presence of obstacles is not critical either: as shown in Fig. 2, dual-task costs increased in the presence of obstacles by a comparable amount in both age groups, and the difference between older and younger subjects therefore remained virtually unchanged (cf. *walk*_*o *_and *walk*). This observation is supported by a statistical analysis of all 13 task combinations from our present and previous study [31], which yielded no significant effect of the factor Obstacles on ARD. The same analysis also yielded no significant effect of the covariate Task Difficulty. Our findings therefore confirm previous reports, according to which ARD is not consistently related to the complexity of walking and non-walking tasks [13,29,30].

The above analysis yielded a significant effect only for the factor Visual Observation: non-walking tasks which required ongoing visual observation led to ARD of more than 8%, while those without such requirements led to near-zero ARD. Our data therefore suggest that visual demand of the non-walking task is critical for the emergence of ARD while walking. This conclusion could explain the conflicting results of previous authors. Some earlier studies combined walking with a complex visual-imagery task; mean dual-task costs in those studies were substantially higher in elderly than in young subjects [29,30]. Other work combined walking with active listening, or with simple reactions to clearly perceptible acoustic or visual signals; in that case, mean dual-task costs were comparable in healthy seniors and in young subjects [27,28,34]. Thus, non-walking tasks with high, but not those with lower demand for visual processing produced ARD, in accordance with our present conclusion. Additional, indirect support for our conclusion is provided by experiments which combined a postural rather than locomotor task with five different non-postural tasks: there, ARD was limited to non-postural tasks with high visual requirements [23]. Our conclusion is also in agreement with the finding that in elderly subjects, body stability is related to visuospatial but not to other cognitive demands [35-37]

To understand why visual demand of the non-walking task is crucial for the emergence of ARD while walking, it should be noted that locomotion is visually demanding as well, since body stability and heading are constantly adjusted with the help of optic flow [38] and visual position cues [39]. The observed deficits could therefore reflect a general problem of seniors to process two sources of visual information at the same time. Indeed, available literature documents several potential reasons for the existence of such a problem. First, old age is characterized by an increase of saccadic latency [40] and a decrease of the useful field of view [41], which could impair seniors' ability to rapidly shift their gaze back and forth between two concurrent tasks. Second, walking becomes increasingly dependent on vision with advancing age [42], possibly due to a reduced proprioceptive and vestibular sensitivity [review in [43,44]]; this could increase the competition between walking and another visually demanding task for visual processing resources [23]. Third, executive functions of the prefrontal cortex decay in old age [review in [18,45]], which could reduce the ability to quickly alternate between the central processing of two visual tasks. Available literature argues against gaze shifting ability as the sole explanation, since substantial ARD was observed even when the non-walking task required visual imagery rather than actual viewing [29,30]. Further research is needed to reliably determine the validity of each above interpretation.

The critical role of vision proposed in the present study is of relevance for many everyday-life scenarios. For example, elderly subjects may have no more problems than younger ones to walk down the street while listening to music, but they may experience difficulties to walk down the street while observing the display in shop windows. In fact, seniors may have a high risk of falling in the latter scenario, since degraded performance on walking with a concurrent visually demanding task is a known predictor of falls in the elderly [46,47]. This differential vulnerability of seniors to scenarios with high versus low visual demand should be taken into account when designing prevention and rehabilitation programs for the elderly.

It should be noted, however, that visual demand may not be the only critical factor for falls in healthy seniors. A range of other predictors not addressed in our study has been identified in literature, such as visual, vestibular, and proprioceptive sensitivity, muscle strength, psychomotor speed, sensorimotor coordination, executive functions, self-efficacy, as well as exposure to slipping and tripping hazards [reviews in [7,9,10,48]]. Additional predictors may exist in seniors suffering from cognitive or sensorimotor dysfunctions: such persons show ARD while walking even if the non-walking task has low visual demand [34,49,50]. Our present findings therefore don't argue against the utility of training programs aimed at those predictors, but rather underline the role of one particular training component.

## Conclusion

In an analysis of 13 combinations between a walking and a non-walking task, we found that dual-task performance is degraded in the elderly for non-walking task which require ongoing visual observation. Such task combinations are common in everyday life, and may therefore contribute to the incidence of falls in seniors. Prevention and rehabilitation programs for the elderly should take this age-related deficit into account, and specifically train participants on task combinations such as walking while adjusting a TV set via remote control, balancing on one leg while reading, standing up and walking while carrying a cup of water [46], etc. Such training is likely to be successful, since seniors' dual-tasking abilities are known to improve by practice [16,32].

## Competing interests

The author declares that they have no competing interests.
